# Mitochondrial dysfunction and neuroinflammatory signaling in a humanized *APP*/*APOE* mouse model of Alzheimer's Disease risk

**DOI:** 10.1002/alz70855_107101

**Published:** 2025-12-25

**Authors:** Mikaila Ann Bantugan, Roberta Diaz Brinton

**Affiliations:** ^1^ University of Arizona, Tucson, AZ, USA

## Abstract

**Background:**

Mitochondrial dysfunction is an early hallmark of Alzheimer's disease (AD), and neuroinflammation is a critical driver of AD pathogenesis. Studies have implicated the cGAS‐STING pathway, a cytosolic DNA sensor and key mediator of innate immunity, in mitochondrial dysfunction, neuroinflammation, and disease progression. The *APOE4* allele, a major genetic risk factor for AD, and the female biological sex may exacerbate mitochondrial dysfunction and neuroinflammation via cGAS‐STING signaling. This study investigated the sex‐ and *APOE‐*specific associations with mitochondrial dysfunction and neuroinflammation via the cGAS‐STING pathway in a genetic risk factor model of AD.

**Method:**

18‐month‐old mice expressing humanized (h) *APP* and h*APOEε3/ε3* (h*APP*/*APOE3*; *n* = 10; 5F) or h*APOE*ε4/ε4 (h*APP*/*APOE4*; *n* = 9; 4F) were included in this study. Bulk RNA sequencing (RNA‐seq) was used to identify differentially expressed genes in RNA isolated from the right hippocampus. Gene Set Enrichment Analysis and Ingenuity Pathway Analysis were used to identify and predict sex and *APOE4* effects upon canonical pathways related to mitochondrial dysfunction, neuroinflammation, and cGAS‐STING signaling. cGAS‐STING pathway activation and inflammatory signaling were further assessed through immunohistochemistry and western blotting. Statistical analyses included 2‐way ANOVA with multiple comparisons in GraphPad Prism and were considered significant at *p* <0.05.

**Result:**

RNA‐seq and pathway analysis revealed increased neuroinflammation, cGAS‐STING signaling, and mitochondrial‐related dysfunction in female h*APP/*h*APOE4* mice compared to male h*APP*/h*APOE4*. In contrast, comparisons between female and male h*APP/*h*APOE3* carriers showed fewer differences in neuroinflammatory or mitochondrial‐related pathways. While sex differences were evident, *APOE* genotype alone did not have a strong effect. However, there was a greater sex‐*APOE*‐genotype interaction effect on neuroinflammation, mitochondrial‐related dysfunction, and the cGAS‐STING signaling. Notably, mitochondrial‐related genes were more dysregulated in female h*APP*/*hAPOE4 compared* to male h*APP/*h*APOE3* mice.

**Conclusion:**

Our findings indicate consistent sex differences in neuroinflammatory and mitochondrial pathways in this mouse model. *APOE* genotype effects were evident in neuroinflammatory signatures, particularly in females. Specifically, elevated cGAS‐STING signaling in female hAPP/h*APOE4*s may drive a neuroinflammatory response, highlighting a potential role in increased AD risk for women during midlife aging. Together, these results highlight the importance of considering both biological sex and genotype effects when investigating the role of neuroinflammatory mechanisms and mitochondrial dysfunction in AD pathogenesis.

